# Attenuation of municipal landfill leachate through land treatment

**DOI:** 10.1186/2052-336X-12-12

**Published:** 2014-01-07

**Authors:** Maryam Pazoki, Mohammad Ali Abdoli, Abdolreza Karbassi, Naser Mehrdadi, Kamyar Yaghmaeian

**Affiliations:** 1 Graduate Faculty of Environment, University of Tehran, Tehran, Iran; 2 Department of Environmental Health Engineering, School of Public Health, Tehran University of Medical Sciences, Tehran, Iran

**Keywords:** Land treatment, Leachate, Municipal solid waste, Slow rate irrigation, Contaminant

## Abstract

The treatment of municipal landfill’s leachate is considered as one of the most significant environmental issues. In this study a laboratory experiment was conducted through land treatment, achieving an efficient and economical method by using Vetiver plant. Moreover, the effects of land treatment of leachate of municipal landfills on the natural reduction of organic and inorganic contaminants in the leachate after the pre-treatment in the Aradkouh disposal center are invested. Three pilots including the under-investigation region’s soil planted by Vetiver plant, the region’s intact soil pilot and the artificial composition of the region’s soil including the natural region’s soil, sand, and rock stone are used. The leachate, having passed its initial treatment, passed through the soil and to the pilot. It was collected in the end of the pilots and its organic and inorganic contaminants were measured. However, the land treatment of leachate was conducted in a slow rate at various speeds. According to the results, in order to remove COD, BOD5, TDS, TSS, TOC the best result was obtained in the region’s soil planted with Vetiver plant and at the speed of 0.2 ml per minute which resulted 99.1%, 99.7%, 52.4%, 98.8%, 94.9% removal efficiencies, respectively. It also can be concluded that the higher the organic rate load is, the lower the efficiency of the removal would be. In addition, EC & pH were measured and the best result was obtained in the region’s soil planted with Vetiver plant and at the speed of 0.2 ml/min.

## Background

One of the most recruited methods to treat waste materials and their disposal is sanitary landfills. This method is used in many countries around the world. Researchers have shown that between 40 to 80% of municipal solid waste (henceforth MSW) is disposed of in developed countries whereas this rate reaches 60 to 90% in developing countries [1].

Leachate micro-organisms can be either broken down or absorbed via the process of biological uptake [2]. This process significantly depends on the establishment of microbial populations as a response to the contaminants’ loading. It is indispensable to investigate the actual pollution plumes to gain efficient insights into the complicated framework to foresee the fate of the contaminants [3].

Due to Tehran’s waste analysis about 68.8% of the solid waste is biodegradable materials which lead to produce huge amounts of leachate with the high organic loads [4,5]. This issue was studied in previous studies which show leachate also contains high volume of inorganic and organic compounds in two phases of suspended or dissolved [6,7]. Hence, leachate is known as hazardous liquid can easily infect the surface water and groundwater [8]. Moreover, as an efficient disposal alternative, leachates are sometimes used for irrigation. This method is appropriate for the polishing of the pre-treated leachate [9].

Appropriate treatment of leachate consequently is regarded as a daunting challenge [10]. The occasional existence of highly concentrated heavy metals makes the biological treatment very difficult due to its toxic effects on microbes [11].

It is possible that leachates contain colossal amounts of organic matter in the forms of biodegradable and biorefractory carbon, nitrogen (bio-nitrogen, ammonia, and nitrate nitrogen), heavy metals and etc. [12].

Two systems namely soil and water are known as the natural treatment systems. When the leachate is on the land surface, under controlled conditions, to achieve a certain level of filtration and treatment through physical, chemical and bio processes in the matrix of water-soil-plant, the soil systems are suggested. Moreover, less energy consumption is needed in the land treatment methods rather than the filtration methods which are mostly due to moving and spreading the leachate [13].

In addition, to achieve the best efficiency among the various irrigation methods, regards to the characteristics of the land treatment with leachate, slow irrigation system is presented. Besides, it leads to high removal of contaminants, BOD_5_ and depositions.

In the land treatment method the pre-treatment process such as screening and primary sedimentation leads to remove suspended solid which prevents damage to irrigation facilities and machinery [14].

Mainly, leachate contains heavy metals. Consequently, a crucial characteristics of plants in phytoremediation needs to be hyper-accumulation. Such type of vegetation is selected due to the physical potential to tolerate and assimilate toxic substances, growing rate, depth of its roots, and capability to degrade or bio accumulate the contaminants in its roots, branches and leaves [15,16].

Using plants in two environments of soil (purification, refinement, preservation, and sustaining soil, sediments) and contaminated waters in previous studies is well accepted [17]. In phyto-remediation methods, one of the significant issues is choosing the kind of the plants. However, plants can lead to water and soil contamination removal and also degrade the bio contaminants. Moreover, they can filter, trap, absorb and stabilize the heavy metals.

Vetiver (Chrysopogonzizanioides) is known as a successfully permanent grass to decline the contaminations due to the mentioned details. It originates from Indian peninsula and for the first time it was used by the World Bank in India [17]. Using Vetiver as a treatment for sewage and leachate was a start point of a great novelty and show the extraordinary capability of plants. In additions, it leads to a green, natural, simple and practical treatment method with low expenses.

The purpose of this study is to evaluate the efficiency of leachate land treatment with the help of Vetiver plant to reduce the COD, BOD_5_, TSS, TDS and the existing TOC in the leachate in municipal landfills leachate after the pre-treatment stage. Moreover, the above mentioned factors against the current standards are compared and discussed.

## Methods

This research presents the results of a laboratory study in a pilot scale which soil profile as a biological filter to attenuation of organic and inorganic contaminants from landfill’s leachate was examined after pre-treatment stage. However, land treatment of leachate in Aradkouh’s landfills has been chosen as the study area.

### Landfill’s characteristics

Aradkouh’s landfill is on the 25^th^ km of Tehran-Qom road and is used as a disposal facility for more than 40 years. More than 8000 ton solid waste is transported to Aradkouh landfill every day which leads to generate leachate about 637500 liters per day. The underground waters level is very low and the wells have a low rate of discharge between 0.5 to 3 liters per second of salty water.

### Raw leachate’s characteristics

The location of sampling effects on the type of leachate. So, fresh leachate is sampled which is needs to sample in the waste discharge place. Usually, these places are dangerous and very hard to take the samples. Otherwise, leachate passes a long way every day and absorb huge amount of contaminants and finally get mixed with the old leachate which leads to lose the initial characteristics. Moreover, the characteristics of generated leachate in Kahrizak’s landfill are given in Table 1.

**Table 1:** Raw leachate’s characteristics in Aradkouh’s landfill

**Parameter**	**Standard methods [**[18]**]**	**Range**	**Average ± S.D**
COD (mg^-1^)	5220-COD	40000-70000	50000 ± 2400
BOD_5_ (mg^-1^)	5210-BOD_5_	20000-30000	27000 ± 1700
TDS (mg^-1^)	2540-TDS	16500-18000	17000 ± 500
TSS (mg^-1^)	2540-TSS	20300-26200	22000 ± 4000
TOC (mg^-1^)	2310-TOC	16500-20000	18000 ± 1600
EC (ms/cm)	2510-EC	27.3-33.3	30.6 ± 3.3
pH	4500-*H*+	4.8-5.1	5 ± 0.3

### The pilot’s characteristics

**Figure 1:**
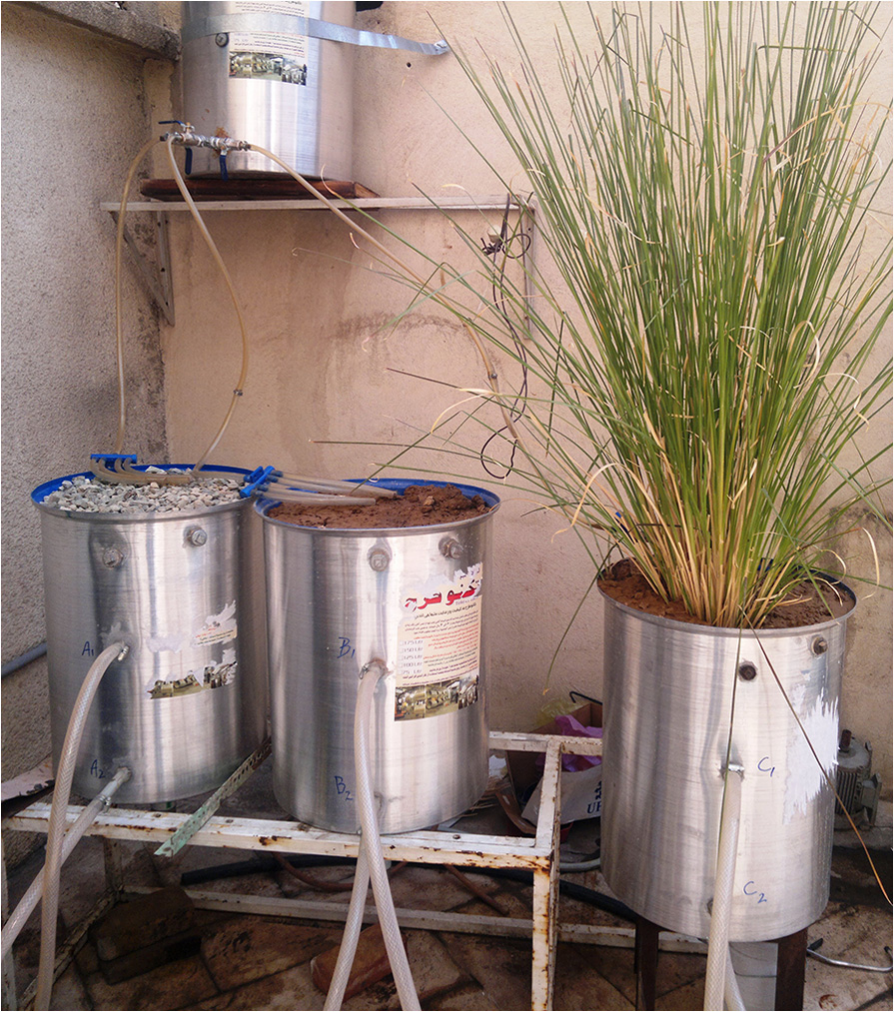
Stages leachate movements from storage to pilots.

In general, three pilots were used and their charecteristics are shown in Table 2. Three various scenarios are conducted to treat the leachate in three pilots. In the first pilot (pilot A), an artificial arrangement soil was used where (from down to top) the first layer is the region soil and the second is sand and rock-stone. On the other hand, the second pilot (pilot B) the natural form of the local soil is used whereas in the third one (pilot C) the local’s natural soil planted by Vetiver is used (Figure 1).

**Table 2:** The pilot’s characteristics

**Tools**	**Number**	**Shape**	**Dimensions**	**Description**
Pilot	3	Cylinder	Dimeter: 70 cm	The pilots were filled with the soil up to the 90 cm of their height
			Height: 120 cm	
Mushy screen	3	Circle	Dimeter: 72 cm	at each 30 cm of the pilots, a pipe was implemented in a steep fashion to get the leachate out
Footstool for installing the pilot	3	Circle	Dimeter: 72 cm	In the bottom of each pilot, a holed metal plate was used for drainage purposes
Reservoir	1	Cylinder	Volume: 75 lit	with three output valves
Footstool for tank installation	1	Cylinder	Dimeter: 100 cm	-
			Height: 200 cm	

### Soil analysis

Physical characteristics of soil such as bulk density, moisture, etc. which are determined according to the standard method of ASTM are presented in Table 3.

**Table 3:** The results of soil studied in Aradkouh’s landfill

**Bulk density**	**K (cm s − 1)**	**ω%**	**pH**	**EC (ms/cm)**	**TDS (g/lit)**
1.86	1.56 × 10^-6^	13	8.2	10.01	5.2

As can be indicated, the analysis of the sample soil including the soil texture and composition are given in Table 4.

**Table 4:** Composition of soil studied

**Composition**	**Test method (ASTM, 2001)**	**Measured value (%)**
Clay and gravel percentage	ASTM-D422	63.62
Sand percentage		33.59
Silt percentage		2.79

### Initial preparation and irrigation

Irrigation technique for forming the structure of soil was conducted for six weeks. The soil underwent wet and dry periods. After that, the output leachate from anaerobic lagoons with a retention time of one month is replaced for irrigation. To achieve the slow rate irrigation leachate sprayed in three rates (0.2, 0.6 and 1 ml/min). However, the process is done at a certain filtration rate and at specific time intervals according to the of slow rate irrigation’s form.

### Laboratory analysis

For running the laboratory tests, the sampling was conducted twice a week. Moreover, to prevent anaerobic conditions the pilots are irrigated with three days interval between irrigations. The samples from the pilots were gathered three times at each rate of 0.2, 0.6 and 1 ml/min, respectively. After that, the output sewage liquids were tested to estimate the COD, BOD_5_, TDS, TSS, TOC and EC. To determine the level of reduction done by soil and plant the results were compared to the pre-treatment stage. It should be mentioned that, the experiments were conducted according to the APHA standard [18]. In addition, to avoid any change in pH values while pH-meter is used due to the CO_2_ evolution, pH was determined immediately after the sampling (pH Tutor, Mfg. by: Eutech Instruments). All the experiments were carried out three times and the mean values are used in this report.

### Statistical analysis

To analysis the results the Duncan test was used. Duncan test can be applied when the number of samples is more than three. In this test, the significant level of (α = 0.05) is assumed.

## Results and discussion

### pH, conductivity and TDS

In this section, the results of the experiments which were conducted in various rates and heights or the three considered pilots are presented and discussed in Tables 5, 6 and 7. In each 30 cm from the top to down of each pilots an output sample was tested which we name them O_1_, O_2_ and O_3_. It should be mentioned that the input of the pilots was the output of pre-treatment section.

**Table 5:** The results of the experiment at the rate of 0.2 ml/min

**Parameter**	**Input**	**Pilot A**			**Pilot B**			**Pilot C**		
	**Average**	**O**_**1**_	**O**_**2**_	**O**_**3**_	**O**_**1**_	**O**_**2**_	**O**_**3**_	**O**_**1**_	**O**_**2**_	**O**_**3**_
EC (ms/cm)	8700	8340	8270	6712	7345	5900	4670	6780	5630	4123
pH	6.13	6.51	7.87	7.12	7.6	7.6	7.9	7.14	7.01	7.02
TDS (mg/l)	4540	4360	4200	3360	3810	3080	2300	3500	2930	2160

**Table 6:** The results of the experiment at the rate of 0.6 ml/min

**Parameter**	**Input**	**Pilot A**			**Pilot B**			**Pilot C**		
	**Average**	**O**_**1**_	**O**_**2**_	**O**_**3**_	**O**_**1**_	**O**_**2**_	**O**_**3**_	**O**_**1**_	**O**_**2**_	**O**_**3**_
EC (ms/cm)	8512	8005	7912	6634	7623	5834	4934	6891	5345	3798
pH	6.8	6.8	7.3	7.7	6.9	7.2	7.7	6.8	7.4	7.6
TDS (mg/l)	4430	4250	4136	3332	3960	3100	2450	3667	2900	2316

**Table 7:** The results of the experiment at the rate of 1 ml/min

**Parameter**	**Input**	**Pilot A**			**Pilot B**			**Pilot C**		
	**Average**	**O**_**1**_	**O**_**2**_	**O**_**3**_	**O**_**1**_	**O**_**2**_	**O**_**3**_	**O**_**1**_	**O**_**2**_	**O**_**3**_
EC (ms/cm)	8576	8367	8100	7111	7789	7083	5902	7498	6127	5101
pH	6.6	6.7	6.7	7.1	6.8	7.3	7.4	7.1	7.2	7.4
TDS (mg/l)	4480	4320	4212	3785	4120	3751	2919	3928	3307	2685

Due to the Tables 5, 6 and 7 the rate of EC decline in various rates for all the pilots was determined. The best efficiency was obtained at the medium rate (0.2 ml/min) at pilot C for the sample O_3_. Moreover, the amount of TDS was decreased along the soil profile. However, the maximum reduction rate was obtained at the medium rate of 0.2 ml/min at pilot C for the sample O_3_. This can be defined by passing the leachate throw the various layer of the soil, biomasses are being formed which lead to the adsorption of some ions by the soil and decrease the TDS.

With regards to the achieved P-value, the amount of EC and TDS are 0.015 and 0.044, respectively. According to the assumed α = 0.05, the p-value is less than α which implies that with varying the flow rate and the pilots cause negligible changes to amounts of EC and TDS.

### COD & BOD_5_

Tables 8, 9, 10 present the COD and BOD_5_ values which were determined due to various rates in the three studied pilots.

**Table 8:** The results of the experiment at the rate of 0.2 ml/min

**Parameter**	**Input**	**Pilot A**			**Pilot B**			**Pilot C**		
	**average (mg/l)**	**O**_**1**_	**O**_**2**_	**O**_**3**_	**O**_**1**_	**O**_**2**_	**O**_**3**_	**O**_**1**_	**O**_**2**_	**O**_**3**_
COD (mg/l)	3840	3560	1120	156	2010	534	82	834	301	35
COD removal (%)	-	7.3	71	96	47.7	86.1	97.9	78.3	92.2	99.1
BOD_5_ (mg/l)	1370	1210	456	54	712	132	14	291	91	4
BOD_5_ removal (%)	-	11.7	66.7	96.1	48	90.4	99	78.8	93.4	99.8
BOD_5_/COD	0.36	0.34	0.41	0.35	0.35	0.25	0.17	0.35	0.30	0.11

**Table 9:** The results of the experiment at the rate of 0.6 ml/min

**Parameter**	**Input**	**Pilot A**			**Pilot B**			**Pilot C**		
	**average (mg/l)**	**O**_**1**_	**O**_**2**_	**O**_**3**_	**O**_**1**_	**O**_**2**_	**O**_**3**_	**O**_**1**_	**O**_**2**_	**O**_**3**_
COD (mg/l)	3700	3680	1900	467	3360	1080	345	1020	512	243
COD removal (%)	-	0.6	48.7	87.4	9.2	70.8	90.7	72.4	86.2	93.4
BOD_5_ (mg/l)	1320	1270	558	101	979	274	61	354	143	35
BOD_5_ removal (%)	-	3.8	57.7	92.4	25.8	79.2	95.4	73.2	86.16	97.4
BOD_5_/COD	0.36	0.35	0.29	0.22	0.29	0.25	0.18	0.35	0.28	0.14

**Table 10:** The results of the experiment at the rate of 1 ml/min

**Parameter**	**Input**	**Pilot A**			**Pilot B**			**Pilot C**		
	**average (mg/l)**	**O**_**1**_	**O**_**2**_	**O**_**3**_	**O**_**1**_	**O**_**2**_	**O**_**3**_	**O**_**1**_	**O**_**2**_	**O**_**3**_
COD (mg/l)	3800	3756	2843	696	3667	1993	583	1437	809	435
COD removal (%)	-	1.2	25.2	81.7	3.5	47.6	84.7	62.2	78.7	88.6
BOD_5_ (mg/l)	1330	1306	874	126	1243	589	96	493	256	59
BOD_5_ removal (%)	-	1.8	34.3	90.5	6.5	55.7	92.8	63	80.8	95.6
BOD_5_/COD	0.35	0.35	0.31	0.18	0.34	0.3	0.17	0.34	0.32	0.14

**Figure 2:**
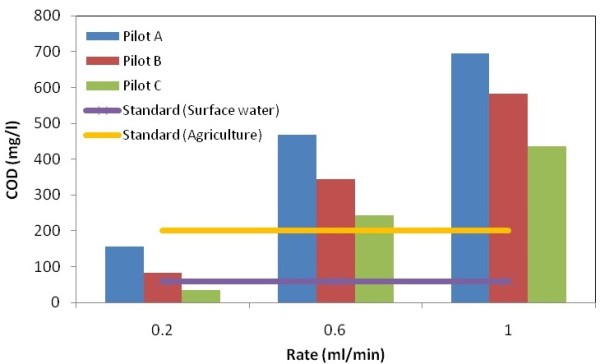
COD values due to various rates.

**Figure 3:**
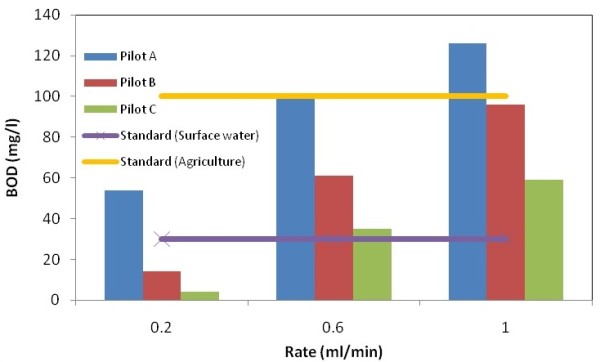
BOD5 values due to various rates.

The output COD and BOD_5_ values due to various rates in the three studied pilots are shown in Figures 2 and 3 which were compared with standards discharging to surface waters, agricultural purposes and irrigation.

With respect to the Tables 8, 9 and 10, the variation of COD and BOD_5_ values due to different rates for each samples are illustrated. Moreover, the special trends are shown and discussed in following. The best efficiency was reached at the medium rate (0.2 ml/min) at the pilot C for the sample O_3_. Moreover, the output results at the speed of 0.2 ml/min at the pilot C for the sample O_3_ meets the both standards discharging to surface waters and agricultural purposes and irrigation.

Along the soil profile the amounts of COD and BOD_5_ were declined. Some parts of particulate COD were decreased by the mechanism of sedimentation and colloidal material adsorbed and then deposited. Some parts of organic materials were reduced by anaerobic decomposition mechanism. BOD_5_/COD ratio was decreased, because microorganisms that were present in the biofilm layer consumed organic matter, also biodegradable organic matter was decomposed thus this ratio is reduced.

In addition, the most elimination rate is founded in the pilot A at the first 30 cm (O_1_), all the three flow rates. However, as much as the soil aggregation gets finer the retention time and the elimination efficiency increased more.

As can be seen, if the organic loading gets more intense (increase the input flow rate), the percentage of removal efficiency would decreases due to the declination of the hydraulic retention time which leads to descend the rate of reduction.

In pilot C in addition to the natural soil zone, the plant growth with the spray roots is effective in the elimination of leachate pollutants. The plant growth regards to the Rhizosphere which is the gathering place of microorganisms and the level of the biological activities are high, with the coexistence between the microorganisms and the absorption of plant roots, lead to more elimination of the leachate pollutants.

The p-values for the COD and the BOD5 are 0.28 and 0.411, respectively. However, it can be concluded that due to varying the flow rates and the pilots the amounts of COD and BOD5 are fluctuated.

### TSS & TOC

Tables 11, 12, 13 show the results of TSS and TOC which were measured for the outputs of the three pilots due to the various speed ratios.

**Table 11:** The results of the experiment at the rate of 0.2 ml/min

**Parameter**	**Input**	**Pilot A**			**Pilot B**			**Pilot C**		
	**average (mg/l)**	**O**_**1**_	**O**_**2**_	**O**_**3**_	**O**_**1**_	**O**_**2**_	**O**_**3**_	**O**_**1**_	**O**_**2**_	**O**_**3**_
TSS (mg/l)	680	380	152	16	212	87	11	230	112	8
TSS removal (%)	-	44.1	77.7	97.7	68.8	87.2	98.4	66.2	83.5	98.2
TOC (mg/l)	860	730	426	67	602	365	57	532	108	44
TOC removal (%)	-	15.1	50.5	92.2	30	57.6	93.4	38.1	87.4	94.9

**Table 12:** The results of the experiment at the rate of 0.6 ml/min

**Parameter**	**Input**	**Pilot A**			**Pilot B**			**Pilot C**		
	**average (mg/l)**	**O**_**1**_	**O**_**2**_	**O**_**3**_	**O**_**1**_	**O**_**2**_	**O**_**3**_	**O**_**1**_	**O**_**2**_	**O**_**3**_
TSS (mg/l)	710	415	178	44	336	75	29	340	53	18
TSS removal (%)	-	41.6	74.9	93.8	52.7	89.4	95.9	52.1	92.5	97.5
TOC (mg/l)	845	742	510	87	738	465	78	770	398	70
TOC removal (%)	-	12.2	39.6	89.7	12.7	45	90.8	8.9	52.9	91.7

**Table 13:** The results of the experiment at the rate of 1 ml/min

**Parameter**	**Input**	**Pilot A**			**Pilot B**			**Pilot C**		
	**average (mg/l)**	**O**_**1**_	**O**_**2**_	**O**_**3**_	**O**_**1**_	**O**_**2**_	**O**_**3**_	**O**_**1**_	**O**_**2**_	**O**_**3**_
TSS (mg/l)	692	456	216	77	401	102	56	369	98	38
TSS removal (%)	-	34.1	68.8	88.9	42.1	85.3	91.9	46.7	85.8	94.5
TOC (mg/l)	860	750	580	123	760	430	113	738	412	99
TOC removal (%)	-	12.8	32.6	85.7	11.6	50	86.9	14.2	52.1	88.5

The measured values of TSS for the samples due to different speed ratios are illustrated in Due to the Tables 11, 12, 13, the best efficiency was achieved at the medium rate of 0.2 ml/min in the sample of O_3_ at pilot C. Moreover, TOC along the profile of soil was decreased and the maximum reduction caused at the medium speed ratio of 0.2 ml/min at sample O_3_ at pilot C. To sum up, the best efficiency was obtained at the pilot C, sample O_3_ whilst the average amounts of TSS and TOC removal were %98.2 and %94.9.

The solid suspended materials are separated before the other materials caused by the flowing throw the soil. A proportion of TSS is eliminated by the screening and sedimentation mechanism and another proportion of fine, soluble and colloidal materials are adsorbed. However, due to the low flow rate the sedimentation is increased. It should be mentioned that, in the systems with rapid penetration most of the solid materials are separated in the surface of the soil. Therefore, it would be possible that the wastewater materials blocking the surface. Hence, drainage systems are suggested.

As can be seen in the above tables, growth in the load of organic materials leads to decrease the elimination efficiency which is caused by increasing the retention time.

As can be seen, in Table 14 a brief literature review of attenuation of landfill leachate with compared to the results of this study is given.

**Table 14:** Literature data of land treatment techniques for reduction of organic material

**Operation condition**	**Results**	**Authors/date**
Landfill leachate, leachate irrigation of woodland, full scale	Leachate concentrations of up to 1500 mg BOD5/l and 300 mg NH4 C-N/l, have been spray irrigated to woodland at appropriate loadings that show no detrimental effects on the irrigated vegetation;	Cornwall County Council [19].
Pilot-scale, land treatment system, treated wastewater from the stabilization pond system of the latex factory	The average removal efficiency of TKN, NH3-N, Org-N, BOD5 and sulfate for tropical carpet grass unit were 92, 97, 61, 88 and 52%, for water convolvulus unit were 75, 80, 43, 41 and 30%, and for control unit were 74, 80, 41, 31 and 28%, respectively.	Thongnuekhang V. and Puetpaiboon. U. [20].
Landfill leachate as irrigation water for tree and vegetable, full scale	The effects of landfill leachate on the growth of tree and vegetable crops were studied with 5, 10, 20 and 40% leachate dilutions than in the non-leachate control. Leachate-treated soil had elevated levels of electrical conductivity, total-, ammonia) and nitrate-N, exchangeable Na and P.	Wong M. H. and Leung C. K. [21].
Young synthetic acetogenic phase landfill leachate, at two hydraulic loading rates (HLR).	The results presented suggest that the HLR of leachate into soil arrays contributes to significant differences in the fate of the landfill leachate parameters (phenol, copper, and zinc) earlier	Kamenthren Govender [22].
Three-year field study, intensive leachate irrigation of two willow varieties	Two willow varieties were tested and four irrigation regimes in sixteen 400-m2 plots. The willow plants did not react negatively, despite very high annual loads of nitrogen, chloride and other elements.	Aronsson P. Dahlin T. Dimitriou I. [23].
Landfill leachate, leachate irrigation of grass and willows, full scale	leachate input (400 m3 per month per hectare in average) did not result in excessive accumulation of salts, heavy metals, or nutrients, which could negatively affect soil properties and plant growth	Justin M. Z. Zupanc M. [24].
Pilot plant scale, Landfill leachate, land treatment, slow rate irrigation, three rate 0.2, 0.6 and 1 ml/min	in order to remove COD, BOD5, TDS, TSS, TOC the best result was obtained in the region’s soil planted with Vetiver plant and at the speed of 0.2 ml per minute which resulted 99.1%, 99.7%, 52.4%, 98.8%, 94.9% removal efficiencies, respectively	This study

## Conclusions

In this research the capability of land filtration and treatment in natural reduction of organic and inorganic contaminants existing in the leachate after the pre-treatment stage was studied. Based on comparison of the results a few points can be concluded which are presented as follows.

1. Land treatment of leachate has a significant positive effect on COD removal and pH stabilization. Due to the achieved results of pilot C (Sample O_3_) at the rate of 0.2 ml/min the maximum reduction efficiency including COD, BOD_5_, TSS & TOC at 99.1%, 99.7%, 98.2% & 94.9% were obtained, respectively.

2. The higher the organic rate load is, the lower the efficiency of the removal would be. In lower hydraulic loads, the reduction rate of organic and inorganic contaminant due to the longer hydraulic retention time is more.

3. Using Vetiver plant in land treatment and filtration of leachate increases the efficiency. It also plays a key role in the contaminant reduction at an appropriate rate (to remove organic and inorganic contaminant, pre-treatment is essential which leads to increase the expenses).

4. This filtration technique compared to the advanced filtration methods, only needs to transmit the leachate from output of the initial sedimentation system, spreading and spraying it on the surface requires energy. Moreover, it needs less mechanical facilities. Also, it requires lower and easier levels of maintenance.

## Competing interests

The authors declare that they have no competing interests.

## Authors’ contributions

All authors contributed to the manuscript. All persons listed as authors have read, contributed to preparing the manuscript and attest to the validity and legitimacy of the data and its interpretation, and agree to its submission to Iranian Journal of Environmental Health Science & Engineering. No person more than the authors listed have contributed significantly to its preparation. All authors read and approved the final manuscript.
